# Editorial: Animal biomechanics: application of biomedical engineering to veterinary sciences for animal healthcare, volume II

**DOI:** 10.3389/fvets.2026.1810150

**Published:** 2026-03-05

**Authors:** Mauro Malvè, Rocío Fernández-Parra

**Affiliations:** 1 Institute of Advanced Materials and Mathematics (INAMAT2), Public University of Navarre, Pamplona, Spain; 2 Research Networking in Bioengineering, Biomaterials & Nanomedicine (CIBER-BBN), Madrid, Spain; 3 Facultad de Veterinaria y Ciencias Experimentales, Universidad Catolica de Valencia San Vicente Martir, Valencia, Spain

**Keywords:** 3D printing, animal biomechanics, biomaterials, finite element simulation, gait analysis, motion analysis, orthopedic, tissue engineering

Biomedical engineering has increasingly demonstrated its value beyond human medicine, yet its application to veterinary science remains an exciting and evolving frontier. Building on the foundations laid in the first volume of this Research Topic, Volume II brings together a diverse and impactful set of contributions that illustrate how engineering principles, experimental techniques, computational modeling, and data-driven methods can meaningfully advance animal health. The 13 articles collected here highlight both methodological innovations and practical applications across species, anatomical systems, and health domains.

Within the field of experimental and clinical biomechanics, Shin et al. present a finite element analysis to evaluate the biomechanical performance of different plate placements in canine elbow arthrodesis. Specifically, caudal, medial, and lateral plate configurations are compared in terms of stress distribution and create stability. Their results indicate that plate position has a substantial influence on load transfer and mechanical behavior of the arthrodesis construct, highlighting the relevance of computational modeling for optimizing surgical planning and implant positioning in veterinary orthopedics. Casas-Alvarado et al. investigate the surface thermal response of peripheral nerve blocks in dogs undergoing trauma or orthopedic surgery, shedding light on analgesic monitoring under clinical conditions. Underberg et al. use time-of-flight magnetic resonance imaging to achieve non-contrast enhanced visualization of equine foot vasculature in a cadaver model, demonstrating a promising imaging approach. Day et al. compare polyethylene cable vs. stainless steel cerclage wire in a canine fracture model, providing biomechanical insight relevant to orthopedic repair. Wang, Zhang et al. contribute two complementary studies on large avian species, reporting on head and neck movement characteristics and on morphological parameters of vertebrae in domestic geese and ducks, respectively, with implications for comparative anatomy, motion analysis, and veterinary ergonomics. Guevar et al. present a comparative biomechanical analysis of monocortical and bicortical polyaxial screw-rod fixation strategies in canine lumbar stabilization, and Kim et al. describe a modified laparoscopic-assisted percutaneous gastropexy technique in dogs, exemplifying surgical innovation and minimally invasive approaches.

This volume also presents advancements in personalized veterinary devices and computational approaches. Sutter et al. present a cadaveric study of patient-specific 3D-printed nasopharyngeal stents in dogs, highlighting the potential of additive manufacturing for customized veterinary implants. Yu et al. apply deep learning to ultrasonographic data to classify canine chronic kidney disease, illustrating the promise of artificial intelligence in diagnostic workflows. Huart et al. employ statistical shape modeling to characterize the geometric morphology of the canine femur, tibia, and patella, offering quantitative reference data that may support clinical decision-making, implant design, and surgical planning. These studies collectively demonstrate how computational modeling, machine learning, and additive manufacturing converge to enable more precise, individualized, and effective interventions in veterinary practice.

Educational tools and clinical biomechanical strategies are also addressed in this volume. Lobo Moraes et al. introduce SimuVet, a preliminary simulator for epidural anesthesia training in dogs, demonstrating how simulation platforms can enhance veterinary education and skills acquisition in controlled and safe environments. Sun et al. investigate *ex vivo* biomechanical interactions of combined extra- and intracapsular stabilization techniques for cranial cruciate ligament deficiency in dogs, contributing to the understanding of stabilization biomechanics and exploring additional biomechanical measures that inform clinical orthopedics and rehabilitation. These studies illustrate the importance of translating fundamental biomechanical knowledge into practical tools for veterinary training and patient care.

Taken together, the articles in Volume II illustrate the range and depth of how biomedical engineering is being applied across veterinary disciplines. From imaging and implant design to computational diagnostics, motion analysis, and educational technologies, this Research Topic demonstrates both technological diversity and translational relevance. At the same time, the volume underscores that many questions remain open—particularly those that lie at the intersection of quantitative model validation, clinical implementation, and species-specific biomechanics. The findings presented here also highlight the need for interdisciplinary collaboration, integrating veterinary clinicians, biomedical engineers, computer scientists, and data analysts to address complex health challenges.

We thank all the authors for their contributions to this Research Topic. We hope that this Research Topic not only enriches the field of veterinary biomechanics but also promotes further collaboration between veterinary clinicians and engineers. By promoting such interdisciplinary partnerships, this research strongly aligns with the One Health framework, which recognizes the interconnection of human, animal, and environmental health. Knowledge and methodologies developed in human biomechanics can, in turn, be applied to animals, improving veterinary care and outcomes. The insights gained from these studies can inform better clinical interventions, enhance animal welfare, and generate findings that may translate back to human health.

